# Retraction: Lakhani et al. Beneficial Role of HO-1-SIRT1 Axis in Attenuating Angiotensin II-Induced Adipocyte Dysfunction. *Int. J. Mol. Sci.* 2019, *20*, 3205

**DOI:** 10.3390/ijms221810081

**Published:** 2021-09-18

**Authors:** International Journal of Molecular Sciences Editorial Office

**Affiliations:** MDPI, St. Alban-Anlage 66, 4052 Basel, Switzerland; ijms@mdpi.com

The journal retracts the article “Beneficial Role of HO-1-SIRT1 Axis in Attenuating Angiotensin II-Induced Adipocyte Dysfunction” [1], cited above.

Following publication, concerns were brought to the attention of the publisher regarding the figures. The microscopy image for one of the experimental groups in Figure 4A was duplicated, appearing in Figure 1A of another published paper [2]. The authors state this was due to carelessness. Additionally, the actin panel in Figure 2D [1] was similar to Figures 2A and 6E of another published paper [3]. The actin panel in Figure 2B [1] was similar to that in Figure 2 of another published paper [4].

The *International Journal of Molecular Sciences* Editorial Office has taken the decision to retract the paper. The editor-in-chief approved this retraction.

The authors agree with the retraction.
